# Impact of free delivery policy on utilization of maternal health services in county referral hospitals in Kenya

**DOI:** 10.1186/s12913-017-2376-z

**Published:** 2017-06-21

**Authors:** John Njuguna, Njoroge Kamau, Charles Muruka

**Affiliations:** 1 Mukurwe-ini sub-County Public Health Office, Mukurwe-ini, Kenya; 2 HEADS Alliance, Kwale County, Kenya; 3 Environmental Health and Allied Services, Kisumu County, Kenya

**Keywords:** Free delivery policy, Kenya, maternal health

## Abstract

**Background:**

Kenya has a high maternal mortality rate. Provision of skilled delivery plays a major role in reducing maternal mortality. Cost is a hindrance to the utilization of skilled delivery. The Government of Kenya introduced a policy of free delivery services in government facilities beginning June 2013. We sought to determine the impact of this intervention on facility based deliveries in Kenya.

**Methods:**

We compared deliveries and antenatal attendance in 47 county referral hospitals and 30 low cost private hospitals not participating in the free delivery policy for 2013 and 2014 respectively. The data was extracted from the Kenya Health Information System. Multiple regression was done to assess factors influencing increase in number of deliveries among the county referral hospitals.

**Results:**

The number of deliveries and antenatal attendance increased by 26.8% and 16.2% in county referral hospitals and decreased by 11.9% and 5.4% respectively in low cost private hospitals. Increase in deliveries among county referral hospitals was influenced by population size of county and type of county referral hospital. Counties with level 5 hospitals recorded more deliveries compared to those with level 4 hospitals.

**Conclusion:**

This intervention increased the number of facility based deliveries. Policy makers may consider incorporating low cost private hospitals so as to increase the coverage of this intervention.

## Background

In 2013, there was an estimated 289,000 maternal deaths globally with sub-Saharan Africa accounting for 62% of these deaths [1]. The fifth millennium development goal aims to reduce the maternal mortality ratio by 75% between 1990 and 2015. Kenya is one of the countries classified as having made insufficient progress towards attaining this goal [2].Kenya has a maternal mortality rate of 488 deaths per 100, 000 births. For Kenya to attain the fifth millennium development target, it needs to have a maternal mortality rate of 147 deaths per 100,000 live births [3]. One strategy essential to reducing the high maternal mortality rates is to ensure that all births are managed by skilled health professionals. World Health Organization advocates for universal skilled birth attendance [4]. In low income settings, an estimated three quarters of neonatal deaths and maternal deaths occur outside hospital [5].

Kenya is located in the East African region and has an estimated population of 44.86 million. It was recently reclassified as a lower-middle income country with a gross national income per capita of 1290 USD. It has a life expectancy of 62 years and 45.9% of the population live in poverty [6]. Kenya’s Human Development Index value in 2013 was 0.535 and it was ranked 147 out of 187 countries [7]. Majority of health services in Kenya were devolved in 2013 and are currently run by a total of 47 county governments [3]. The health service delivery in Kenya is organized across six levels of care, beginning at the community level and continuing through the primary care services, which include dispensaries (level 2) and health centers’ (level 3). These are followed by county referral health services (level 4 and 5). Level 6 consists of the national referral health services [8]. Majority of county referral hospitals are level 4 hospitals. This is because level 5 hospitals are few as majority were formerly called provincial general hospitals serving several counties prior to devolution of health services.

Kenya recorded an increase in the proportion of facility based deliveries from 44% in 2008 to 61% in 2014 [9, 10]. This increase has been partly attributed to the free maternal care policy introduced in June 2013 [3]. This policy enables pregnant women access free maternity services in all public health facilities. In addition, the user fees of Kenya Shillings 10 (0.1 US Dollars) and 20(0.2 US Dollars) normally charged in dispensaries and health centers’ respectively were also abolished. The current arrangement is that the health facilities provide these services and are later reimbursed by the Ministry of Health headquarters as per the number of deliveries conducted. The rates are 2500 shillings (28.7 US Dollars) per birth at level 2 and 3 facilities and 5000 shillings (57.5 US Dollars) at level 4, 5 and 6 facilities. These rates cover all types of deliveries and are paid directly to the facilities. Also, no fees are charged for antenatal and post-natal care up to six weeks after delivery or for referrals made due to pregnancy related complications [3]. This study looked at the impact of this intervention on facility based delivery in county referral hospitals and among low cost private hospitals for comparison.

## Methods

The study analyzed data on antenatal attendance and facility based deliveries. These were extracted from the Kenya Health Information System website (https://hiskenya.org). This is based on the free and open- source web-based District Health Information Software (DHIS2) [11]. This is run by the division of health information, Ministry of Health Kenya. The division has health information and records officers working in health facilities. They get various reports generated by health facilities, collate and upload them into this website. We looked at data for one year pre-intervention and one year post-intervention for the 47 county referral hospitals and compared them to that of 30 not-for profit private hospitals not participating in the free maternity program. The pre-intervention period was from June 2012 to May 2013 while the post intervention period was from June 2013 to May 2014. Majority of these private hospitals are run by religious organizations under the auspices of the Kenya Episcopal Conference-Catholic Secretariat and Christian Health Association of Kenya respectively.

The study utilized the integrated reproductive health, HIV/AIDS, malaria, tuberculosis and nutrition report commonly called MOH 711. Data were extracted from the safe delivery section. This section has the number of maternal deaths and births. Total births were derived by adding the number of normal deliveries, caesarian section, breech delivery and assisted vaginal delivery. Antenatal attendance is listed as new attendance and re-attendance. These two were added to give total antenatal attendance. Standard multiple regression was used to assess the ability of poverty, total fertility rate, population and type of county referral hospital to predict the increase in number of deliveries in 2014 among county referral hospitals. Data on poverty levels per county were extracted from the Kenya Economic Report, 2013 [12]. Data on population per county were extracted from the national census reports [13]. Data on total fertility rate per county were extracted from the Kenya Demography and Health Survey 2014 [10]. Preliminary analyses were conducted to ensure no violations of the assumptions of normality, linearity, multicollinearity and homoscedasticity. Analysis was done using Ms. Excel and SPSS version 16.

This study was not reviewed and approved by an institutional review board. Ethical approval was not necessary as the study utilized data sets available in a public website. The data on the Kenya health information system is normally de-identified to protect patient confidentiality and privacy.

## Results

Antenatal care attendance increased by 16.2% in 2014 among the county referral hospitals. This was from 247,251 in 2013 to 287,312 in 2014. The number of deliveries increased from 147,262 in 2013 to 186,688 in 2014 representing an increase of 26.8%. Number of deliveries increased in all county referral hospitals except one which recorded a 4.3% decline (Table 1). The highest increase recorded was 79.8%. Level 5 hospitals recorded a higher increase compared to level 4 hospitals with a mean increase of 1498 deliveries compared to 613. Among the private hospitals, antenatal attendance declined by 5.4%. This was from 91,413 in 2013 to 86,471 in 2014. Deliveries declined by 11.9%. This was from 36,531in 2013 to 32,179 in 2014.

**Table 1:** Increase in Deliveries in County Referral Hospitals 2014

Name of County	% increase in Deliveries	% increase in antenatal attendance	Population of County
BARINGO	45.4	19.2	555,561
BOMET	7.1	12.3	585,072
BUNGOMA	2.5	42.3	1,375,063
BUSIA	6.9	12.6	743,946
ELGEYO MARAKWET	14.6	21.9	369,998
EMBU	1.9	21.1	516,212
GARISSA	64.6	13.2	623,060
HOMA BAY	40.1	15.3	958,791
ISIOLO	47.2	44.2	143,294
KAJIADO	45.5	30.8	687,312
KAKAMEGA	1.1	17	1,660,651
KERICHO	6.4	8.5	758,339
KIAMBU	0.2	17.5	1,623,282
KILIFI	42.7	30.8	1,109,735
KIRINYAGA	0.5	−14.9	528,054
KISII	0.6	−10.4	1,152,282
KISUMU	14.3	−6.3	968,909
KITUI	32.2	34.6	1,012,709
KWALE	59.3	54	649,931
LAIKIPIA	14.4	19.7	399,227
LAMU	22.3	26.6	101,539
MACHAKOS	2.3	35.4	1,098,584
MAKUENI	1.6	19.6	884,527
MANDERA	67.2	27.4	1,025,756
MARSABIT	58.6	9.5	291,166
MERU	2.1	0.5	1,356,301
MIGORI	29.2	15.4	917,170
MOMBASA	2.7	47	939,370
MURANGA	0.2	41.3	942,581
NAIROBI	0.4	35.1	3,138,369
NAKURU	4.3	−3	1,603,325
NANDI	4.2	14.6	752,965
NAROK	50.3	31	850,920
NYAMIRA	0.4	−4.4	598,252
NYANDARUA	0.1	−2.7	596,268
NYERI	0.2	26.5	693,558
SAMBURU	75.3	8.3	223,947
SIAYA	19	−1.1	842,304
TAITA TAVETA	5.5	20.5	284,657
TANA RIVER	71.9	19.3	240,075
THARAKA NITHI	3.5	−8.5	365,330
TRANS NZOIA	2.3	17.3	818,757
TURKANA	88.4	27.7	855,399
UASIN GISHU	1.6	22.7	894,179
VIHIGA	0.5	11.8	554,622
WAJIR	77.5	3.9	661,941
WEST POKOT	67	7.9	512,690

Poverty rate per county, population per county, type of county referral hospital, total fertility rate per county accounted for 37.4% of the variance in increase in number of deliveries in 2014 among county referral hospitals (Adjusted R square = .374, *p* < .001). Only population (beta = .384, *p* = .004) and type of county referral hospital (beta = .424, *p* < .002) were statistically significant (Table 2).

**Table 2:**
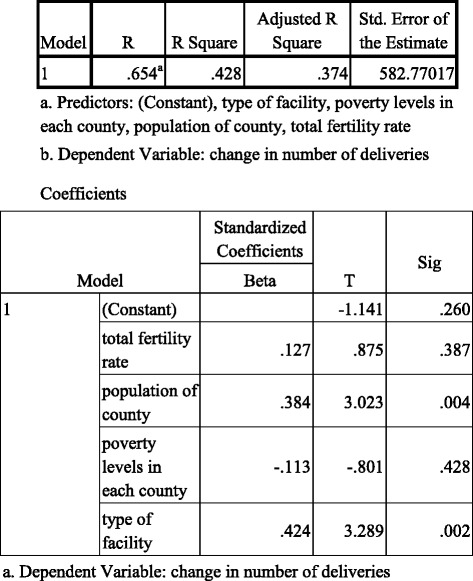
Regression Results

## Discussion

Deliveries and antenatal attendance increased in government health facilities providing free maternity care. On the other hand, deliveries and antenatal attendance declined in low cost private hospitals not providing free maternity care. This may be attributed to pregnant women who previously could not afford to deliver in a health facility utilizing the free maternity services as well as some who may have sought care from private hospitals reverting back to public hospitals. Cost is an important factor in accessing health care in Kenya. In sub-Saharan Africa, the poorest women are over three times more likely to report giving birth at home than the richest women [14]. In Kenya, women from wealthier households and those with health insurance are more likely to deliver in a health facility compared to women from poorer households and those without insurance [15]. Women of low socioeconomic status may have benefited more from this intervention. Prior to the free maternity policy women would be detained in public hospitals after delivery due to non-payment of bills. This became a thing of the past with this intervention [16]. A review by the Ministry of Health found that in the entire country, normal deliveries and those via caesarean increased by 22% and 17% in the financial year 2013/14 compared to 2012/13. Antenatal revisits increased by 13% during the same period [3]. Nepal introduced a free delivery policy in 2009 and an evaluation done 10 months after the policy found that facility based deliveries had increased by 19% [17]. A policy of providing free care for caesareans and under-fives was launched in 2008 in northern Sudan. A review done two years later found that normal deliveries increased by 14% [18]. Ghana introduced a free maternal care initiative in 2008. This was through the provision of health insurance to pregnant women giving them access to comprehensive maternity care, with the exception of ambulance services and post partum family planning counseling. This led to an increase in the number of facility based deliveries from 300,000 in 2007 to about 500,000 in 2011 [19].

Level 5 hospitals registered more deliveries compared to level 4 hospitals. This could be due to the fact that level 5 hospitals are more equipped and more staffed compared to level 4 hospitals. All level 5 and 6 hospitals offer comprehensive obstetric care. Not all level 4 hospitals offer comprehensive obstetric care. A study found that 46.4% of level 4 hospitals offered only basic obstetric care [3]. Antenatal attendance did not increase as much as deliveries. Pregnant women may have preferred to attend lower level health facilities for their antenatal care. These lower level facilities are more likely to be nearer their homes and likely to be less congested compared to county referral hospitals. Antenatal attendance declined slightly in private hospitals compared to deliveries. This could be due to the cost of antenatal services being relatively cheap compared to that of delivery. Some antenatal services may be offered free of charge at low cost private hospitals e.g. immunization. Population size influenced number of deliveries. Counties with high population tend to have a higher number of women of reproductive age compared to less populated counties. This may have translated to higher number of deliveries.

This free delivery policy has increased facility based deliveries. Despite this, an estimated 39% of pregnant women still do not deliver in a health facility [10]. This varies across the counties from 6.6% to 81.7%. In six counties, less than a third of women deliver in a health facility [10]. A study has shown that facility based delivery is influenced by four thematic issues with cost being one of them [20]. These are socio-cultural, perceived benefits, physical accessibility and economic accessibility. Providing free services only partly addressed economic accessibility [20]. Multivariate analysis has also shown that in sub-Saharan Africa, maternal education, parity, rural/urban residence, household wealth, distance to health facility are strongly associated with facility based deliveries [21].In Kenya, only 11% of women cited cost as a reason for not delivering in a health facility. Other factors cited included inaccessible health facilities, lack of transport, and the perception that it is not necessary to deliver in a health facility, and the delivery occurring too fast before one could reach a facility [15]. The number of deliveries in hospitals in Ghana almost doubled a year after introduction of free maternal health services. The number of deliveries later declined. This was attributed to other factors not being addressed e.g. accessibility of health facilities [19].

Other interventions albeit on a small scale may have positively impacted the free maternity program. These include the Beyond Zero campaign launched in January 2014 by the First Lady of the Republic of Kenya with an aim of improving maternal and child health outcomes. A major activity of this campaign has been the provision of a mobile clinic to each county with an aim of providing healthcare to poor and marginalized communities. By April 2015, 22 mobile clinics had been donated. These mobile clinics have provided healthcare for over 30,000 Kenyans as at February 2016 [22]. On the other hand, health workers in all government facilities went on strike for 11 days in December 2013 to protest the devolution of health services [23]. All government hospitals except the two level 6 facilities were closed and patients discharged arbitrarily including expectant women. This adversely affected the free maternity program.

The free delivery policy could be improved for greater impact. It has been argued that increasing demand for services must be accompanied by strategies to ensure that the supply side can cope [24]. A review of the free maternity services found some limitations [3, 15]. These included weak referral system, inadequate staff, amenities and equipment to deal with the increased workload. There were also delays in reimbursing hospitals for delivery services rendered by the national government. Clients also stated that the service was not entirely free with 28% having paid for something. This ranged from various tests, registration, drugs, x-ray services and antenatal care booklet [3].

Deliveries in the private hospitals declined. Involving the private sector may increase the impact of this intervention. The ministry of health has 4077 health facilities. Other public institutions like communities, local authority, armed forces have a total of 433 health facilities. Private institutions and private practice comprise of 3600 facilities, faith based organizations 1063 facilities and non- governmental organizations 327 [25]. Involving the private sector may increase the reach of this intervention and improve on facility based deliveries. However the rates reimbursed per delivery may be deemed inadequate by the private sector with the exception of not for profit health providers. In Kenya some not for profit providers e.g. faith based health facilities provide health care to poor and underserved populations who may not easily access government health facilities. In Ghana, the private sector was involved in providing free maternal health services and 16% of deliveries occurred in the private health facilities [19]. In Nepal, the not-for-profit health facilities e.g. mission hospitals and non-governmental run hospitals were enrolled in the free delivery policy [17].

This study has some limitations. Reporting systems are liable to have missing data due to under reporting and misreporting. Over reporting can also occur as a result of reporting aggregate data and not individual records. However the Kenya Health Information System has inbuilt validation rules and data quality checks. This includes limiting access to those who can enter the data. It also uses a cloud-based central server. This enables any changes made in the system to be available immediately to all users and that the system is available on a 24/7 basis. It also has an option to cater for offline data entry to cater for areas with poor internet connectivity [11]. At the hospital or sub-county level, health information is part of the supportive supervision process. These incorporate on the job training on data quality. Regular data review meetings are convened by the health information and records office. These are normally attended by health managers. Any gaps in data quality are identified and addressed. An evaluation of hospital management information system in 22 Kenyan hospitals in 2012 found constraints in data quality assurance, supportive supervision, financial resources and human resources [26]. It would have been ideal for the study to consider additional baseline years going as far as 2010. This data is not available as between 2008 and 2011, the official health information system in Kenya was the File Transfer Protocol (FTP) and this had its major challenges. The major one was the huge time lag between data generation and reception of the same at the national level [11]. Unavailability of this data means that our study may not account for year to year fluctuation of hospital births and this is a limitation.

There could also be the financial pressure to report more deliveries so as to be reimbursed more. The Ministry of Health headquarters also carries out validation of data submitted for reimbursement purposes. This includes random visits to hospitals to peruse individual patient files and the number of birth notification slips sent to the registrar of births and deaths. However these are limited in scope and coverage.

## Conclusions

This study found that the number of deliveries in county referral hospitals increased following the introduction of the free maternity services. Involving the private sector especially the not-for profit health providers may increase the impact of this intervention.

## Abbreviations

USD: United States Dollar
